# Genetic Diversity in the mtDNA of *Physarum polycephalum*

**DOI:** 10.3390/genes14030628

**Published:** 2023-03-02

**Authors:** Freya Hammar, Dennis L. Miller

**Affiliations:** Department of Biological Sciences, The University of Texas at Dallas, Richardson, TX 75080, USA

**Keywords:** myxomycetes, mitochondrial DNA, RNA editing, cryptogenes, linear mitochondrial plasmid, open reading frames, mitochondrial recombination

## Abstract

The mtDNA of the myxomycete *Physarum polycephalum* can contain as many as 81 genes. These genes can be grouped in three different categories. The first category includes 46 genes that are classically found on the mtDNA of many organisms. However, 43 of these genes are cryptogenes that require a unique type of RNA editing (MICOTREM). A second category of gene is putative protein-coding genes represented by 26 significant open reading frames. However, these genes do not appear to be transcribed during the growth of the plasmodium and are currently unassigned since they do not have any apparent similarity to other classical mitochondrial protein-coding genes. The third category of gene is found in the mtDNA of some strains of *P. polycephalum*. These genes derive from a linear mitochondrial plasmid with nine significant, but unassigned, open reading frames which can integrate into the mitochondrial DNA by recombination. Here, we review the mechanism and evolution of the RNA editing necessary for cryptogene expression, discuss possible origins for the 26 unassigned open reading frames based on tentative identification of their protein product, and discuss the implications to mtDNA structure and replication of the integration of the linear mitochondrial plasmid.

## 1. Introduction

### Mitochondrial DNA Evolution

Although most of the genes of eukaryotic organisms are located on nuclear chromosomes, a small number of genes are located on the mitochondrial DNA (mtDNA) in mitochondria. These genes are necessary for mitochondrial function and biogenesis. They include genes that encode mRNAs for protein subunits of complexes of the ETC (electron transport chain) and mitochondrial ATP synthase involved in the OXPHOS (oxidative phosphorylation) pathway. In addition, some of the genes encode tRNAs and rRNAs necessary for mitochondrial protein synthesis and often genes encoding mRNAs for protein subunits for the mitochondrial ribosomes [1].

It is nearly universally accepted that mitochondria derive from a single endosymbiotic event in which a eubacterium was taken up by either a proto-eukaryote [2] or an archaebacterium [1]. The eubacterium is widely accepted to have been of the class α-proteobacterium and order Rickettsiales based on the sequence similarity and gene organization between *Rickettsia* and the mtDNA of certain protists, exemplified by *Reclinomonas americana* [1]. The gene content and size of the protomitochondrial genome is difficult to predict since contemporary mtDNAs vary extensively in size, structure, and gene content [3,4,5,6,7]. Gene reduction has been a universal result of mtDNA evolution, although the extent of gene loss varies widely among the mtDNAs of animals [5], plants [6], fungi, and protists [7]. Even the number of protein-coding genes in the mtDNA of *R. americana* (69 genes) is greatly reduced relative to *Rickettsia* (834 protein-coding genes), indicating a tendency of gene reduction due to gene loss or to transfer of mitochondrial genes to the nucleus known as endosymbiotic gene transfer (EGT) [1].

The mtDNA of *R. americana* contains 98 genes all of which appear to be of eubacterial origin. These genes include 26 tRNA genes, 3 rRNA genes, and 69 protein-coding genes which are thought to be retained from the original protomitochondrial genome and to constitute one of the mtDNAs with the least gene reduction of any contemporary mtDNA [1].

In this review, we compare the mtDNA of the myxomycete, *P. polycephalum*, to the mtDNA of *R. americana*, focusing on the unique features of *P. polycephalum* mtDNA. We will review what is currently known regarding (1) the unique type of RNA editing required for the expression of some genes on the mtDNA, (2) the origin and function of unique genes found on *P. polycephalum* mtDNA, and (3) the unique transition from a circular to a linear mtDNA in some *P. polycephalum* strains.

## 2. The Mitochondrial DNA of *P. polycephalum*

**Figure 1:**
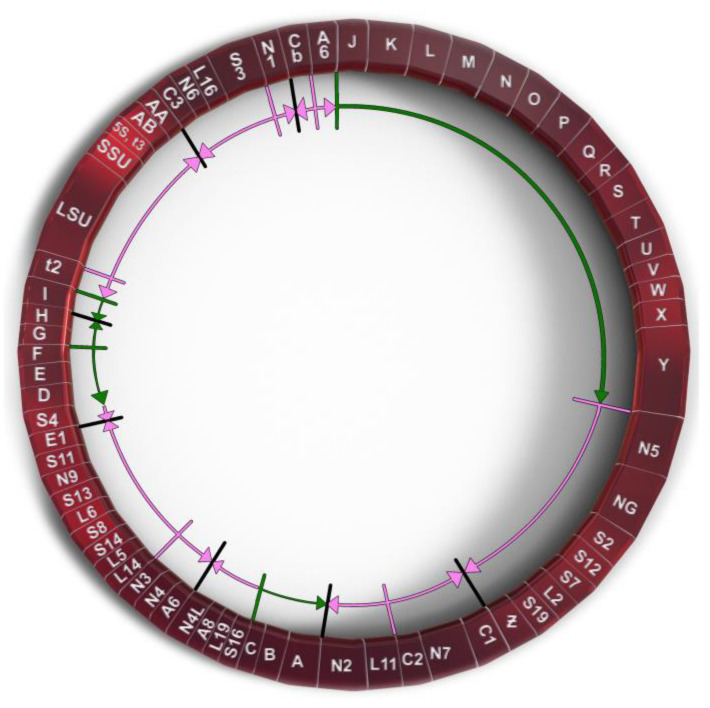
Genetic map of *P. polycephalum*’s mtDNA showing its 72 genes. Gene symbols are as defined in Table 1. Genes which have their direction of potential transcription marked by green arrows are unassigned reading frames. Genes marked with pink arrows show the direction of transcription for classic mitochondrial genes.

*P. polycephalum* is a myxomycete or acellular slime mold and is a eukaryote belonging to a clade whose ancestor diverged early from those of plants, animals, and fungi. The most common mtDNA structure in strains of *P. polycephalum* is circular and contains 72 genes (Figure 1, Table 1). The mtDNA varies in size from 56 to 62 kb, depending on the presence of some small deletions in the mtDNA of some strains [8], reviewed in [9]. This mtDNA is unique in that it is composed of two different types of gene. The first type includes 46 genes which are homologous to genes found on the mtDNA of *R. americana* and together account for 38.5 kb of the mtDNA (red arrows in Figure 1), indicating that they are derived from the common eubacterial ancestor. A second type of potential gene includes 26 significant open reading frames located on 24.3 kb of the mtDNA (green arrows in Figure 1). The 26 unassigned open reading frames are interspersed with the classical, ancestral genes at four positions (group 1, URFs A, B, C; group 2, URFs D–I; group 3, URFs J–Y; group 4, URF Z; Figure 1). Only one ORF (URF Z) is transcribed, the other 25 URFs are not transcribed [10,11].

The 46 genes that are homologous to genes in the mtDNA of *R. americana,* indicating that the *P. polycephalum* mtDNA has retained these genes from the protomitochondrial genome, can be considered ancestral relative to other presumably derived, unique characteristics of the mtDNA. These derived features include (1) a reduction in the tRNA genes (only five tRNA genes remain indicating that the majority of mitochondrial tRNAs are imported from the cytoplasm), (2) the presence of a unique type of RNA editing necessary for the expression of 43 of the classical mtDNA genes (one tRNA gene, one rRNA gene, and one protein-coding gene do not require this editing), (3) the presence of 26 significant but unassigned open reading frames, (4) the presence of nine additional open reading frames in some strains that derive from recombination with a linear mitochondrial plasmid, mF.

### 2.1. Mitochondrial Insertional Cotranscriptional RNA Editing in the Myxomycetes (MICOTREM)

**Figure 2:**
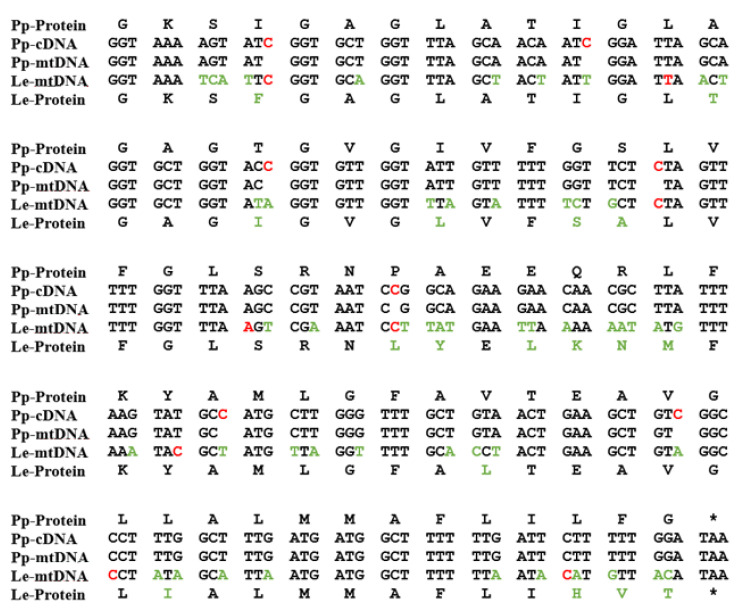
A portion of the atp9 gene on the mtDNA of *P. polycephalum* (Pp-mtDNA) and *Lycogala epidendrum* (Le-mtDNA) aligned with *P. polycephalum* cDNA (Pp-cDNA) and the inferred protein products using the classic genetic code. The alignment shows the editing site distribution and variation in editing site locations in different myxomycetes. Red letters in Pp-cDNA are experimentally determined RNA editing sites in *P. polycephalum.* Red letters in Le-mtDNA are RNA editing sites inferred by alignment. Green letters in Le-mtDNA and Le-Protein show differences between the *P. polycephalum* and *L. epidendrum* sequences. * indicates termination codon.

Probably the most unique derived characteristic of the *P. polycephalum* mtDNA is the RNA editing needed to express 43 of the 47 transcribed genes (recently reviewed in [9] and [12]). This unique type of RNA editing was first identified and characterized by Mahendran et al. [13] in the α subunit of the ATP synthase cryptogene (genes requiring RNA editing to provide genetic information necessary for their expression) in the mtDNA of *P. polycephalum* and later extended to additional cryptogenes in the *P. polycephalum* mtDNA [14,15,16,17,18,19]. This unique type of RNA editing has been designated MICOTREM (Mitochondrial Insertional, Cotranscriptional RNA Editing in Myxomycetes). Currently, MICOTREM has only been found in the mtDNAs of the myxomycetes [20] and produces genetic information lacking in the cryptogenes by inserting nucleotides in RNA relative to the template DNA to create open reading frames in mRNAs and functional RNA structure in tRNAs and rRNAs. These non-templated nucleotide insertions are most commonly single cytidines but can also be single uridines or a subset of the possible dinucleotides (CU or UC, AA, GC or CG, UU, UA). These non-templated insertions are separated by an average of about 25 nucleotides in mRNAs, so that about 4% of the mRNA nucleotides are non-templated. Although the distribution of editing sites appears essentially random, no two insertion sites have been observed closer than nine nucleotides (Figure 2). Overall, 1324 RNA editing sites have been identified in the RNAs produced from the classical, ancestral genes of *P. polycephalum*, 1301 single-nucleotide sites and 23 dinucleotide sites for a total of 1347 non-templated nucleotides added to mitochondrial RNAs [11].

The mechanism of this RNA editing is the co-transcriptional addition of specific nucleotides to the 3′ end of the nascent RNA transcript at editing sites by the mitochondrial RNA polymerase [21,22]. This is in contrast to post-transcriptional mitochondrial insertional editing systems which use antisense guide RNAs (gRNAs) to specify editing site location and insert nucleotides by breaking the RNA backbone at a specific location (RNA endonuclease), adding one or more non-templated nucleotides to the 3′ end of the cleaved RNA (terminal transferase), and then restoring the RNA backbone by RNA ligation (RNA ligase), reviewed in [23]. In *P. polycephalum* this co-transcriptional mechanism consists of the mitochondrial RNA polymerase proceeding along the template DNA strand, adding templated nucleotides during normal transcription until it arrives at an editing site. The polymerase then pauses in transcription, adds a specific non-templated nucleotide or dinucleotide to the 3′ end of the nascent RNA, and then resumes normal transcription using the DNA template to specify nucleotide addition.

The mitochondrial RNA polymerase of *P. polycephalum* is similar to other mitochondrial RNA polymerases that lack MICOTREM RNA editing in that it consists of a single polypeptide with significant homology to several bacteriophage RNA polymerases [24]. How the mitochondrial RNA polymerase identifies the location of an editing site, or how the specific nucleotide or dinucleotide is specified has not been determined. The complexity of this RNA editing argues that genetic information must be required to specify nucleotide identity and location. Rigorous searches for antisense nucleotides with editing site information (gRNAs) have not revealed any candidates. The only repository of the complete mitochondrial genetic information is the fully edited mRNA itself, but it is unclear how this genetic information could be used to specify nucleotide location as a nascent RNA is being transcribed and edited. Although no consensus sequence in the mtDNA around editing sites has been found, Rhee et al. [25] have shown that sequences just upstream and downstream of editing sites are critical for correct editing. Miller, Padmanaban, and Sancar [9] have proposed that RNA editing sites are identified by duplex formation between the fully edited RNA and its antisense DNA template. Bases inserted in the RNA relative to the DNA template by editing would create a nucleotide bulge in the DNA-RNA duplex in which the unpaired RNA base could be flipped out of the duplex without significant disruption of the duplex. This flipped out base could serve as the marker for RNA editing site location. Retention of the edited RNA which is Watson–Crick-based paired with the DNA template, and a displaced non-template DNA strand plectonemically associated with the major groove of the duplex and stabilized via Hoogsteen base pairing would produce a DNA-RNA-DNA triplex. In vivo and in vitro testing of this model are in progress.

A second major unanswered question about MICOTREM editing is how non-templated nucleotides are specified at RNA editing sites. This specificity is clearly necessary since any of the RNA nucleotides can be inserted at RNA editing sites but always the same nucleotide or dinucleotide is inserted at a given RNA editing site. Miller and Miller [22] have shown that the *P. polycephalum* mitochondrial RNA polymerase is able to add random nucleotides to the 3′ end of RNAs in vitro in the presence of complex RNA sequences. Likewise, Sarcar and Miller [26] have shown that T7 RNA polymerase is able to add random ribonucleotides to the 3′ ends of RNAs and DNAs in the presence of complex DNA or RNA sequences. In addition, they demonstrated that T7 RNA polymerase (a member of the super family of single-subunit polymerases including mitochondrial polymerases) can add specific nucleotides to the 3′ ends of DNAs or RNAs in vitro by creating the potential for intramolecular or intermolecular base pairing which creates recessed 3′ ends that can be extended by one or a few nucleotides on the template provided by the extended 5′ end (limited primer extension). This templated activity can occur in the absence of transcription when only a single nucleotide is provided. Whether the *P. polycephalum* mitochondrial RNA polymerase can also add specific nucleotides to the 3′ end of RNAs in vitro by limited primer extension has not been determined.

An alternate way in which the edited nucleotide could be specified is through a binding factor that recognizes the flipped-out nucleotide in an edited RNA/template DNA duplex (see above) and either provides an identical nucleotide triphosphate to the active site of the RNA polymerase which would be added without a template or directly adds the identical nucleotide to the 3′ end of the nascent RNA through a terminal transferase activity. This model would require a specific binding factor for each of the mono- or dinucleotides that can be inserted. Binding of the factor at the editing site might cause the RNA polymerase to pause in transcription to allow the insertion of the non-template nucleotide by either mechanism.

#### Evolution of RNA Editing Site Location

Comparison of RNA editing sites within the same genes of different myxomycetes shows that while they all display MICOTREM editing, the location of RNA editing sites varies relative to conserved regions within analogous genes (Figure 2). In contrast to the conservation of editing sites in the mtDNA of individual myxomycetes, this observation implies an unanticipated dynamic in the location of editing sites over evolutionary time periods and provides insight into the constraints on editing site location and distribution, as well as the mechanism of editing site fixation and elimination. Krishnan et al. [20] compared editing site location in a 452-nucleotide region of the small subunit rRNA among six myxomycetes. Each myxomycete had a similar number of editing sites (eight to ten) which were distributed such that no two editing sites were closer than nine nucleotides and, in each case, restored the conserved sequence of the SSU rRNA. However, these editing sites were distributed in different patterns in the six different RNAs and were located at 29 different sites relative to the conserved sequence of the RNA. In general, the more closely related the myxomycetes, the more editing sites they have at the same location. These variations indicate that editing sites can be created and/or removed over evolutionary time. Analysis of these editing patterns in relationship to established phylogenetic trees confirm that editing sites have been both created and deleted during the evolution of the mtDNA to produce the editing patterns observed in contemporary organisms.

Krishnan et al. [20] described a model of RNA editing site fixation during evolution. This model proposes a mechanism that compensates for random deletions in DNA by inserting a non-templated nucleotide at the analogous location of the RNA. Since this compensation can restore the function of the RNA, this editing site becomes a fixed feature of the expression of the mutated gene. Since these editing sites are created by random deletions, the editing site distribution would be expected to be random except for the constraint that editing sites cannot be closer than 9 nucleotides. This constraint limits the density to which editing sites can accumulate. Simulations of random accumulation of sites constrained by at least 9 nucleotides between editing sites produces patterns of editing similar to those observed in contemporary myxomycete mtDNAs. The saturation density of editing sites under these constraints is an average of about 22 nucleotides between editing sites, a density consistent with the observed density of about 25 nucleotides between editing sites.

Editing site patterns may also be altered by the removal of RNA editing sites. Landweber [27] and Simpson and colleagues [28,29] have proposed retrotranscription as a mechanism of eliminating insertional editing sites. Integration of cDNAs produced from reverse transcription of edited RNAs would remove the deletions in the mtDNA and eliminate the need for a compensating insertion of nucleotides in the RNA.

### 2.2. Unidentified, Untranscribed but Significant Open Reading Frames in the mtDNA of P. polycephalum

A second unique feature of *P. polycephalum* mtDNA is the presence of 26 open readings that do not correspond to any of the genes classically observed on mitochondrial DNAs [8,9]. Most of these reading frames are significantly long (greater than 100 codons), so that they are not likely to have been generated by chance, but with one exception are not transcribed [10,11] The fact that these significant unassigned reading frames (SURFs) remain intact in the absence of the transcription that would provide the selection to maintain open reading frames, implies that they may be recently acquired.

The 26 SURFs are interspersed within the classical genes of the mtDNA in four groups. Group 1 SURFs are designed A, B, and C and would be transcribed counterclockwise in Figure 1 in the order CBA. URFs A and B would code for proteins of 238 and 411 amino acids, respectively. These proteins have transmembrane characteristics consistent with being membrane proteins. However, they do not have significant homology with any protein in GenBank. URF C, a smaller open reading frame, also does not have significant homology to any gene in GenBank.

The Group 2 untranscribed region has six SURFs, two that would be transcribed clockwise in Figure 1 (G and H), and four which would be transcribed counterclockwise in Figure 1 (I, F, E, D). SURFs D, E, H, and I have transmembrane characteristics but none of these SURFs have significant similarity to proteins in GenBank.

The largest group of SURFs is Group 3 which includes SURFs J to Y and covers 18,022 base pairs of the mtDNA. These 16 SURFs would all be transcribed clockwise in Figure 1 and have very little noncoding space between reading frames. These SURFs are predicted to code for proteins ranging in size from 112 amino acids (SURF V) to 724 amino acids (URF Y). SURFs J, K, L, N, O, Q, S, T, and U would code for proteins predicted to have transmembrane features and could be membrane proteins. Most of these SURFs would code for proteins that do not have similarity to any proteins in GenBank; however, several of the proteins predicted to be produced from these SURFs have recently been matched with proteins. SURF N (400 amino acids) and SURF Q (389 amino acids) have similarity to each other and to a hypothetical protein from *Flavobacteriales bacterium* (328 amino acids, GenBank sequence ID: NQX98395.1) recently identified during a metagenomic search of ocean water from the marine abyssalpelagic zone, Pacific Ocean, North Pacific Gyre, Station ALOHA (Leu, A. O., 2020, unpublished). All three hypothetical proteins have a region of similarity of about 200 amino acids starting at 139 amino acids from the N-terminus. SURF R (663 nucleotides, 221 amino acids) has a region of identity to SURF 7 (1098 nucleotides, 366 amino acids) in the mitochondrial mF plasmid (see below). This region of identity in SURF R is 474 nucleotides (158 amino acids) in length starting near the N-terminus. This region of identity is the site of homologous recombination between the circular *P. polycephalum* mtDNA and the mF plasmid (see below). The one SURF with the potential to produce a protein with a known function is SURF Y. SURF Y has the potential to produce a protein 724 amino acids in length. This protein has significant homology to a number of single subunit RNA polymerases from linear mitochondrial plasmids. This RNA polymerase is presumably not the mitochondrial RNA polymerase used to transcribe genes on the mtDNA of *P. polycephalum*, since this SURF is not transcribed, and the encoded RNA polymerase is not produced. (The actual RNA polymerase used to transcribe the mtDNA is encoded in the nucleus and is well characterized [24].) The similarity of the SURF Y amino acid sequence to RNA polymerases on linear mitochondrial plasmids and the identity of the portion of SURF R with SURF 7 of the mF linear mitochondrial plasmid argues that this region of the mtDNA may derive from a linear mitochondrial plasmid or a related bacteriophage with a linear double stranded DNA such as phi 29 [30].

Group 4 consists of one SURF, ORF Z. In contrast to the other unassigned reading frames, ORF Z is transcribed and in a clockwise direction. It is possible that this unidentified ORF is a classical mitochondrial gene but has diverged to an extent that it cannot be identified. However, the absence of MICOTREM RNA editing argues against it being classical and argues for it being recently acquired.

### 2.3. Mitochondrial Open Reading Frames Derived from the mF Mitochondrial Plasmid

A third unique feature of *P. polycephalum* mtDNA is that in some strains of *P. polycephalum* the mtDNA contains an additional nine open reading frames and has a linear, rather than circular, structure. This results from recombination between the linear mF plasmid and the circular mtDNA, resulting in the integration of the mtDNA within the mF plasmid producing a linear 77.3 kb mtDNA with nine additional reading frames [31,32,33].

**Figure 3:**
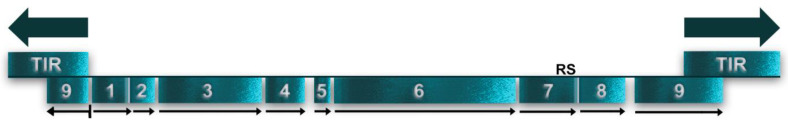
Map of the linear mF plasmid showing significant open reading frames numbered 1 to 9. Arrows indicate the direction of transcription. TIR at the termini indicate terminal inverted repeats. RS indicates the potential recombination site with the mtDNA.

**Figure 4:**
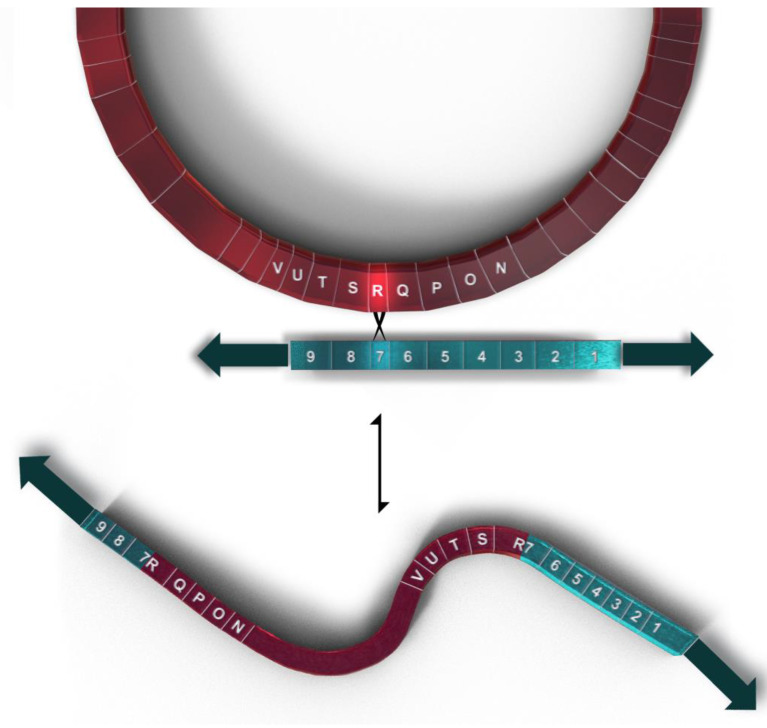
Representation of the recombination between the circular mtDNA (shown in magenta) and the linear mF plasmid (shown in teal) to produce the 77.3 kb linear mtDNA with 81 genes. Genes flanking the recombination site are shown on both plasmid and mtDNA using gene symbols shown in Table 1.

**Figure 5:**
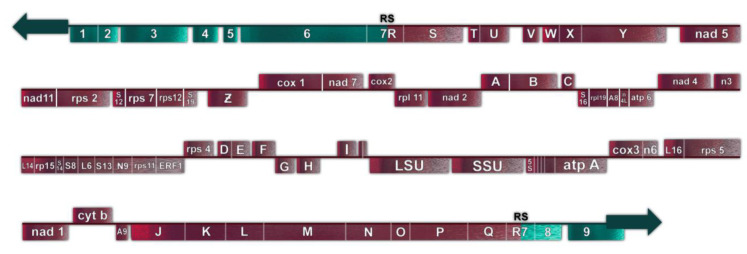
Representation of the 77.3 kb linear mtDNA. Gene symbols are as defined in Table 1. DNA derived from the circular mtDNA is shown in magenta; DNA derived from the linear mF plasmid is shown in teal.

The mF plasmid is a 14,503 base pair linear, double stranded genetic element with terminal inverted repeats (TIRs), located in the mitochondria of some *P. polycephalum* strains (Figure 3). It contains nine ORFs, all oriented in the same transcriptional direction which are transcribed in multiple RNAs [34] (Figure 3). This linear mitochondrial plasmid belongs to a class of structurally and functionally related genetic elements termed invertrons which have linear double stranded DNA genomes with terminal inverted repeats and proteins attached to the 5′ termini which are involved in their replication [30]. Two of the ORFs have been tentatively identified. ORF 9 has significant similarity to a number of B family DNA polymerases [35]. ORF 6 has homology to certain fungal RNA polymerases, at least some of which are located in other linear mitochondrial plasmids. Although currently unassigned, the remaining open reading frames presumably code for proteins involved in autonomous replication, transcription of the plasmid, and integration of the plasmid into mtDNA. SURFs 3 and 8 have transmembrane features and may be membrane proteins. SURF 7 in the mF plasmid has partial homology to SURF R in the mtDNA (see above). This homology is used for homologous recombination which integrates the mtDNA into the plasmid producing a 77.3 kb mF plasmid-mtDNA recombinant with the plasmid inverted repeats as telomeres (Figure 4). This 77.3 kb mtDNA contains 81 genes (Figure 5) and may replicate using the plasmid replication mechanism. On the linear mtDNA, two hybrid genes, ORF R7 (630 bp) and ORF 7R (1761 bp), are produced by the recombination of ORF R (663 bp) and ORF 7 (1098 bp). These hybrid ORFs flank the mtDNA sequences at the boundary of the mF plasmid sequences (Table 1, Figure 4 and Figure 5).

The origin of the mF plasmid is unclear. It apparently does not have any obligate function since it is absent from some strains without any growth or respiration phenotype. Kawano et al. [36] report that strains with the mF plasmid have a mif^+^ (mitochondrial fusion) phenotype which allows mtDNA recombination between heteroplasmic zygotes. Its ability to recombine with mtDNA which anciently derives from eubacterial DNA makes it reminiscent of integrating bacteriophages. Bacteriophage phi29 is a member of the invertron family. It has a 19,285 base pair linear double stranded DNA genome, inverted terminal repeats, 5′ terminal binding proteins, and genes for RNA and DNA polymerases similar to linear mitochondrial plasmids, but in addition has genes for structural and capsid proteins [37]. Mitochondrial DNAs have some characteristics that are more typical of a bacteriophage origin rather than a eubacterial origin. Most dramatically, essentially all mtDNAs are transcribed with a single subunit bacteriophage-like RNA polymerase rather than a typical eubacterial α_2_ββ’σ multisubunit RNA polymerase. The involvement of bacteriophage in the endosymbiont origin of mitochondria is unexplored. Further characterization of the genes encoded in the mF plasmid may provide insights into whether this plasmid has bacteriophage origins. Moreover, the fact that integration of the plasmid occurs in the SURF R region of the mtDNA is further evidence that this region also derives from a mitochondrial plasmid. Further characterization of the SURFs in the mtDNA and linear MF plasmid may strengthen this hypothesis.

## Figures and Tables

**Table 1:** Gene Symbols used in figures.

**Symbol**	**Gene Name**	**Gene Length (bp)**	**ClassicalMt Gene**	**RNA** **Editing**	**Transcription** **Direction**	**URF Feature**
**Circular Mitochondrial DNA Genes**						
**t2**	tRNA^E^ gene	69	C	E	CCW	
	tRNA^M1^ gene	69	C	E	CCW	
**LSU**	Large subunit rRNA gene	2718	C	E	CW	
**SSU**	Small subunit rRNA gene	1814	C	E	CW	
**5S**	5S rRNA gene	96	C		CW	
**t3**	tRNA^M2^ gene	72	C		CW	
	tRNA^K^ gene	72	C	E	CW	
	tRNA^P^ gene	71	C	E	CW	
**AB**	atp B, ATP synthase, subunit B	293	C		CW	
**AA**	atp A, ATP synthase, subunit A	536	C	E	CW	
**C3**	cox 3, cytochrome oxidase subunit 3	762	C	E	CCW	
**N6**	nad 6, NADH dehydrogenase, subunit 6	479	C	E	CCW	
**L16**	rpl 16, large subunit ribosomal protein 16	525	C	E	CCW	
**S3**	rps 3, small subunit ribosomal protein 3	1370	C	E	CCW	
**N1**	nad 1, NADH dehydrogenase, subunit 1	929	C	E	CW	
**Cb**	cob, cyt b, cytochrome b oxidase	1136	C	E	CCW	
**A6**	atp 9, ATP synthase, subunit 9	243	C	E	CW	
**J**	URF J, Unassigned reading frame J	1412			CW	TM
**K**	URF K, Unassigned reading frame K	1062			CW	TM
**L**	URF L, Unassigned reading frame L	1086			CW	TM
**M**	URF M, Unassigned reading frame M	2217			CW	
**N**	URF N, Unassigned reading frame N	1200			CW	TM, Fb
**O**	URF O, Unassigned reading frame O	579			CW	TM
**P**	URF P, Unassigned reading frame P	1509			CW	
**Q**	URF Q, Unassigned reading frame Q	1167			CW	TM, Fb
**R**	URF R, Unassigned reading frame R	663			CW	RS
**S**	URF S, Unassigned reading frame S	1629			CW	TM
**T**	URF T, Unassigned reading frame T	222			CW	TM
**U**	URF U, Unassigned reading frame U	1128			CW	TM
**V**	URF V, Unassigned reading frame V	336			CW	
**W**	URF W, Unassigned reading frame W	393			CW	
**X**	URF X, Unassigned reading frame X	669			CW	
**Y**	URF Y, Unassigned reading frame Y	2172			CW	RNAP
**N5**	nad 5, NADH dehydrogenase, subunit 5	1894	C	E	CW	
**NG**	nad 11/G, NADH dehydrogenase, subunit 11	1017	C	E	CW	
**S2**	rps 2, small subunit ribosomal protein 2	1356	C	E	CW	
**S12**	rps 12, small subunit ribosomal protein 12	496	C	E	CW	
**S7**	rps 7, small subunit ribosomal protein 7	764	C	E	CW	
**Z**	URF Z, Unassigned reading frame Z	352			CW	T
**C1**	cox 1, cytochrome oxidase subunit 1	1719	C	E	CCW	
**N7**	nad 7, NADH dehydrogenase, subunit 7	1065	C	E	CCW	
**C2**	cox 2, cytochrome oxidase, subunit 2	864	C	E	CCW	
**L11**	rpl 11, large subunit ribosomal protein 11	877	C	E	CW	
**N2**	nad 2, NADH dehydrogenase, subunit 2	1407	C	E	CW	
**A**	URF A, Unassigned reading frame A	714			CCW	TM
**B**	URF B, Unassigned reading frame B	1233			CCW	TM
**C**	URF C, Unassigned reading frame C	222			CCW	
**S16**	rps 16, small subunit ribosomal protein 16	525	C	E	CW	
**L19**	rpl 19, large subunit ribosomal protein 19	553	C	E	CW	
**A8**	atp 8, ATP synthase, subunit 8	222	C	E	CW	
**N4L**	nad 4L, NADH dehydrogenase, subunit 4L	275	C	E	CW	
**A6**	atp 6 ATP synthase, subunit 6	708	C	E	CW	
**N4**	nad 4, NADH dehydrogenase, subunit 4	1383	C	E	CCW	
**N3**	nad 3, NADH dehydrogenase, subunit 3	374	C	E	CCW	
**L14**	rpl 14, large subunit ribosomal protein 14	354	C	E	CW	
**L5**	rpl 5, large subunit ribosomal protein 5	485	C	E	CW	
**S14**	rps 14, small subunit ribosomal protein 14	265	C	E	CW	
**S8**	rps 8, small subunit ribosomal protein 8	422	C	E	CW	
**L6**	rpl 6, large subunit ribosomal protein 6	467	C	E	CW	
**S13**	rps 13, small subunit ribosomal protein 13	539	C	E	CW	
**N9**	nad 9, NADH dehydrogenase, subunit 9	475	C	E	CW	
**S11**	rps 11, small subunit ribosomal protein 11	721	C	E	CW	
**E1**	ERF 1, edited reading frame 1	652	?	E	CW	
**S4**	rps 4, small subunit ribosomal protein 4	790	C	E	CCW	
**D**	URF D, Unassigned reading frame D	267			CCW	TM
**E**	URF E, Unassigned reading frame E	612			CCW	TM
**F**	URF F, Unassigned reading frame F	441			CCW	
**G**	URF G, Unassigned reading frame G	738			CW	
**H**	URF H, Unassigned reading frame H	597			CW	TM
**I**	URF I, Unassigned reading frame I	390			CCW	TM
**mF Plasmid Genes**						
**1**	URF 1, mF unassigned Reading frame 1	696			LTR	
**2**	URF 2, mF unassigned Reading frame 2	492			LTR	
**3**	URF 3, mF unassigned Reading frame 3	1923			LTR	TM
**4**	URF 4, mF unassigned Reading frame 4	708			LTR	
**5**	URF 5, mF unassigned Reading frame 5	357			LTR	
**6**	URF 6, mF unassigned Reading frame 6	3393			LTR	RNAP
**7**	URF 7, mF unassigned Reading frame 7	1101			LTR	RS
**8**	URF 8, mF unassigned Reading frame 8	930			LTR	TM
**9**	URF 9, mF unassigned Reading frame 9	1644			LTR	DNAP
**Linear mtDNA Hybrid Genes Produced by Recombination**						
**R7**	URF R7, hybrid unassigned Reading frame R7	630			LTR	
**7R**	URF 7R, hybrid unassigned Reading frame 7R	1131			LTR	

C = classical mtDNA genes; E = cryptogenes requiring MICOTREM RNA editing for their expression; CW = clockwise transcription or potential transcription in Figure 1; CCW = counterclockwise transcription or potential transcription in Figure 1**;** LTR = genes transcribed from left to right; T = a transcribed significant open reading frame (URF Z) which has not been identified as a classical mtDNA gene; TM = Open reading frames whose inferred proteins have potential transmembrane domains; RS = Open reading frames containing the mF plasmid recombination site; Fb = Open reading frames with homology to a hypothetical protein from *F. bacterium;* RNAP = Open reading frames with domains similar to single subunit mitochondrial/phage-type DNA directed RNA polymerases; DNAP = Open reading frames with domains similar to single subunit DNA directed DNA polymerases.

## Data Availability

Data sharing not applicable.
